# Home Nursing of Insured Persons

**Published:** 1922-08

**Authors:** Irene M. Hett

**Affiliations:** M.G.G.I., Hon. Secretary, The Ranyard Nurses. 25, Russell Square, W.C.1.


*To the Editor of* The Hospital and Health Review.

### Home Nursing of Insured Persons.

*To the Editor of* The Hospital & Health Review.

Dear Sir,—Reference is made under the above heading in your last issue to the arrangement with the Queen Victoria's Jubilee Institute whereby certain of the Approved Societies provide home Nursing for their members. We should like to point out that the same position holds good for all members living in the many areas in London where Ranyard Nurses are at work. To simplify the arrangements, the Queen Victoria's Jubilee Institute have very kindly undertaken to receive the returns sent in by the Ranyard Nurses, and to carry through the negotiations with the Approved Societies, in the same way as they do for their own affiliated Nursing Associations.

Yours, &c.,
Irene M. Hett, M.G.G.I.,
Hon. Secretary, The Ranyard Nurses.
25, Russell Square, W.C.1.

Will a reader of The Hospital and Health Review please exchange for " Nursing Mirror " weekly ? Address " Mirror," care of Editor.

